# Surface Structure Reformulation from CuO to Cu/Cu(OH)_2_ for Highly Efficient Nitrate Reduction to Ammonia

**DOI:** 10.1002/advs.202404194

**Published:** 2024-08-09

**Authors:** Jin Li, Qiuling Jiang, Xiujing Xing, Cuilian Sun, Ying Wang, Zhijian Wu, Wei Xiong, Hao Li

**Affiliations:** ^1^ Key Laboratory of Novel Biomass‐Based Environmental and Energy Materials in Petroleum and Chemical Industry Key Laboratory of Green Chemical Engineering Process of Ministry of Education Hubei Key Laboratory of Novel Reactor &Green Chemical Technology School of Chemistry and Environmental Engineering Wuhan Institute of Technology Wuhan 430205 China; ^2^ Advanced Institute for Materials Research (WPI‐AIMR) Tohoku University Sendai 980–8577 Japan; ^3^ School of Applied Chemistry and Engineering University of Science and Technology of China Hefei 230026 China; ^4^ Changchun Institute of Applied Chemistry Chinese Academy of Sciences Changchun 130022 China; ^5^ Chemistry Department University of California Davis CA 95616 USA

**Keywords:** Cu(OH)_2,_ CuO, NO_3_RR, structural and phase changes

## Abstract

Electrochemical conversion of nitrate (NO_3_^−^) to ammonia (NH_3_) is a potential way to produce green NH_3_ and remediate the nitrogen cycle. In this paper, an efficient catalyst of spherical CuO made by stacking small particles with oxygen‐rich vacancies is reported. The NH_3_ yield and Faraday efficiency are 15.53 mg h^−1^ mg_cat_^−1^ and 90.69%, respectively, in a neutral electrolyte at a voltage of ‐0.80 V (vs. reversible hydrogen electrode). The high activity of the electrodes results from changes in the phase and structure during electrochemical reduction. Structurally, there is a shift from a spherical structure with dense accumulation of small particles to a layered network structure with uniform distribution of small particles stacked on top of each other, thus exposing more active sites. Furthermore, in terms of phase, the electrode transitions from CuO to Cu/Cu(OH)_2_. Density functional theory calculations showed that Cu(OH)_2_ formation enhances NO_3_‐ adsorption. Meanwhile, the Cu(OH)_2_ can inhibit the competing hydrogen evolution reaction, while the formation of Cu (111) crystal surfaces facilitates the hydrogenation reaction. The synergistic effect between the two promotes the NO_3_‐ to NH_3_. Therefore, this study provides a new idea and direction for Cu‐based oxides in electrocatalytic NH_3_ production.

## Graphical abstract

A high‐performance small particle stacked spherical CuO catalyst for NO_3_RR is developed. Benefiting from the Cu/Cu(OH)_2_ structure formed during the electrolytic reduction process, it has excellent NO_3_^−^ reducing properties. Where Cu(OH)_2_ enhanced the adsorption of nitrate ions and the inhibition of the competitive HER reaction, and the presence of Cu facilitated the hydrogenation reaction.

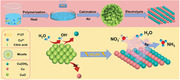

## 1 Introduction

Ammonia, as an indispensable feedstock for food production and industrial development, has a market size of a staggering 175 million tonnes and a market value of $67 billion, representing ≈ 5 percent of the total chemical market value. In addition, as a high energy density energy carrier, ammonia is at the heart of the global hydrogen economy (known as Hydrogen 2.0).^[^ ^1^, ^2^, ^3^ ^]^ However, the conventional Haber‐Bosch ammonia synthesis process has harsh reaction conditions and must be carried out at high temperatures and pressures, leading to disadvantages such as high energy consumption, cost and CO_2_ emissions. As a result, an increasing number of researchers are working to develop a green, efficient, and sustainable route to ammonia synthesis. Possible routes that have been identified include nitrogen‐fixing enzyme catalysis, multiphase catalysis, electrocatalysis, and photocatalysis.^[^ ^4^, ^5^ ^]^ Among these, electrocatalytic ammonia synthesis (including nitrogen reduction reaction (NRR) and nitrate reduction reaction (NO_3_RR)) is a research hotspot and is considered as a promising alternative method.^[^ ^6^ ^]^ In the NRR reduction reaction, the reactant nitrogen (N_2_) exists in large quantities in the air (78%), which is easy to obtain with good economic benefits, but under ambient conditions, the high dissociation energy of the N≡N triple bond (941 kJ mol^−1^) and the low solubility of N_2_ in water (0.66 mmol L^−1^) make it difficult to break the triple bond and achieve a high ammonia yield. In contrast, nitrate (NO_3_^−^) as a nitrogen source has a much lower dissociation energy (the N≡O triple bond dissociation energy of NO_3_^−^ is 240 kJ mol^−1^), it also has much higher solubility in water and it is easier to use water as a hydrogen source for the reduction reaction.^[^ ^7^, ^8^, ^9^, ^10^, ^11^, ^12^, ^13^, ^14^, ^15^ ^]^ NO_3_RR not only bypasses the difficult N≡N activation and complex gas‐liquid‐solid interfacial reaction, but also shows higher Faraday efficiency (FE) and ammonia yield, making it easier to achieve industrialization and scale‐up. At the same time, NO_3_RR uses nitrate as the raw material for the reaction, which not only can solve the ecological problems of nitrate accumulation in water and nitrogen cycle caused by industrial and agricultural production, but also can turn waste into treasure by converting nitrate into more value‐added ammonia.^[^ ^16^, ^17^, ^18^, ^19^ ^]^

The synthesis of ammonia from NO_3_RR is a complex process involving the transfer of 9 protons and 8 electrons (NO_3_^−^ + 9 H^+^ + 8 e^−^→NH_3_ + 3 H_2_O). This process inevitably produces by‐products such as nitrogen‐oxygen ions and dinitrogen, accompanied by the hydrogen evolution reaction (HER) side reaction.^[^ ^20^, ^21^, ^22^, ^23^ ^]^ Therefore, to further improve the efficiency of NO_3_RR ammonia synthesis, the development of catalysts with high activity, high selectivity, and high stability is necessary. Cu‐based catalysts are considered to be one of the most promising materials for the NO_3_RR reaction due to their effective inhibition of HER and similar *d*‐orbital energy levels to those of the LUMO π* molecular orbitals of nitrates.^[^ ^24^, ^25^, ^26^ ^]^ However, due to the strong adsorption of NO_3_RR intermediates (e.g., NO_2_^−^ and NO), pure copper electrodes typically suffer from low stability and poor NH_3_ selectivity. To overcome these limitations and improve the activity and selectivity of copper‐based catalysts, various strategies have been developed, including transition metal alloying, metal oxides, and noble metal doping.^[^ ^27^, ^28^ ^]^ Among them, Cu‐based oxides are capable of potential‐dependent oxidation state evolution (e.g., reduction to metallic Cu) and structural transformation (e.g., atomic rearrangement) under NO_3_RR conditions, thus promoting NO_3_RR.^[^ ^21^, ^29^, ^30^, ^31^, ^32^, ^33^, ^34^, ^35^, ^36^ ^]^ Therefore, researchers expect to be able to use CuO as a cathode material for electrodes – it has a polymorphic structure, and it is stable, inexpensive, and easy to handle. By controlling the NO_3_RR conditions and performing controlled in situ reconstruction, the ideal elemental valence and morphological structure can be formed to change the electronic structure of the electrode surface and improve the selectivity, optimize the adsorption energy barriers, and improve the kinetic reaction to achieve the highly efficient synthesis of ammonia.

Herein, we synthesized spherical CuO catalyst stacked with small particles of oxygen‐rich vacancies by a simple sol‐gel method to be used for the synthesis of NH_3_ via NO_3_RR. The characterization data of the CuO spheres after NO_3_RR showed a morphological change from a spherical shape with small particle stacking to a layer‐stacked reticulation structure. This structure exposed more active sites and greatly facilitated the mass/charge transfer process in the electrocatalytic process. The phase was transformed from initial CuO to Cu/Cu(OH)_2_. Electrochemical measurements indicate that the multivalent (0, +2) composition of the Cu/Cu(OH)_2_ structure enhances the electron transfer for reactions at the electrode surface. Additionally, the hydrogenation reaction in the NO_3_RR process was facilitated by Cu (111) in Cu/Cu(OH)_2_. Density functional theory (DFT) calculations suggested that Cu(OH)_2_ (021) significantly reduced the energy barrier for NO_3_^−^ adsorption in the NO_3_RR process, making it a spontaneous and energetically favorable process. The reaction energy for this first adsorption step was exothermic at −0.62 eV. Cu(OH)_2_ was also found to be effective in inhibiting the competing HER. The synergistic interaction between both Cu and Cu(OH)_2_ together promoted NO_3_RR. The outstanding physical‐chemical properties of CuO/CC demonstrated its superior catalytic performance in the electrocatalytic conversion of NO_3_ to NH_3_. At −0.80 V (vs reversible hydrogen electrode, RHE) with 0.5 M Na_2_SO_4_ + 0.05 M NO_3_^−^ as the electrolyte, the NH_3_ yield on CuO/CC was as high as 15.53 mg h^−1^ mg_cat_^−1^ (or 913.53 umol h^−1^ mg_cat_^−1^), while maintaining a high FE of 90.69%.

## 2 Results and Discussion

### 2.1 Synthesis and Structural Characterizations of Catalyst

**Figure 1:**
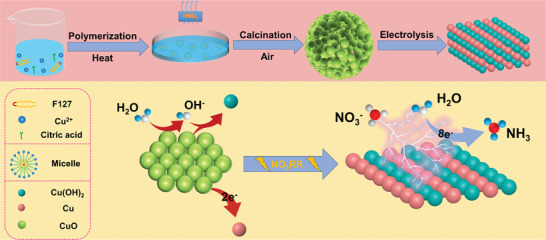
The CuO synthesis route and its reaction process at the cathode.

The CuO electrode materials were synthesized using a sol‐gel method, as shown in **Figure** **1**. Nitric acid was added to create acidic conditions to inhibit the ionization of citric acid and rapid polymerization of the precursors during the sol stage of the synthesis process. After gradually raising the temperature to 120 °C, the nitric acid evaporated (boiling point of nitric acid 86 °C) during the process. As a result, the acid inhibition gradually weakened, and the Cu compound precursor began to polymerize slowly. After drying, calcination was carried out under air atmosphere to obtain spherical CuO consisting of small particles stacked with oxygen‐rich vacancies (see the Experimental Section for detailed steps).

**Figure 2:**
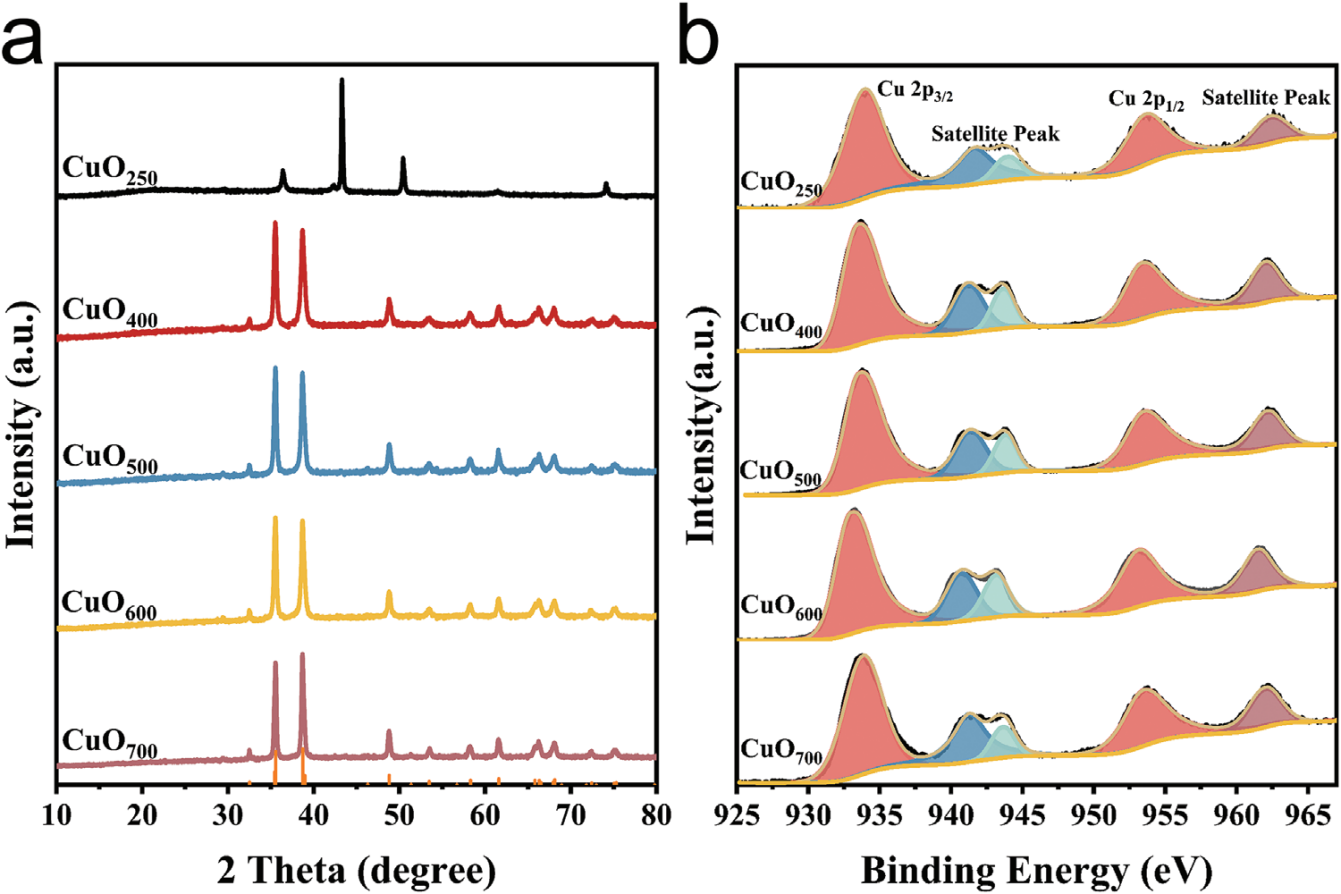
CuO at different calcination temperatures: a) XRD and b) high‐resolution XPS spectra of Cu 2p.

**Figure 3:**
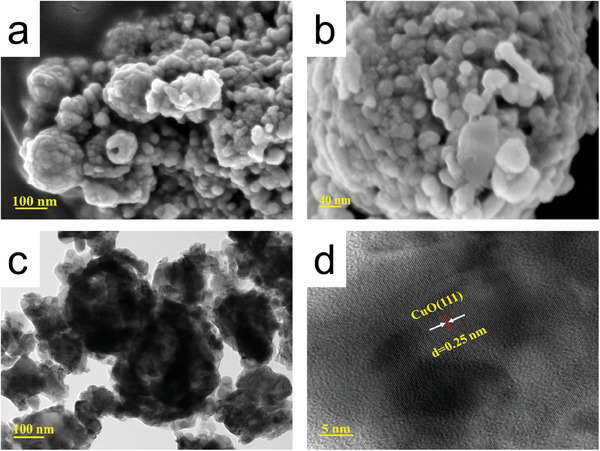
a,b) SEM, c) TEM, and d) HR‐TEM images of CuO_500_.

The prepared CuO samples were characterized by SEM, TEM, XRD, and XPS for morphology, composition, and valence distribution. First, different samples were analyzed by XRD to understand the crystal structure information of the prepared samples. **Figure** **2**a shows that, apart from CuO_250_, all other samples at different temperatures exhibited a single CuO (PDF#89‐5895) crystal structure without any other phases. The characteristic peaks at 38.7° and 35.5° corresponded to CuO (111) crystal faces. Comparison of samples at different calcination temperatures showed that the characteristic peak of copper oxide became sharper and more crystalline with increase in temperature. The characteristic peaks of CuO_250_ were not clearly visible due to the lower calcination temperature and poor crystallinity. Additionally, Figure 2a showed slightly protruding carbon peaks between 15° and 30°, indicating that CuO_250_ retained part of its carbon structure at low calcination temperatures. To further verify the presence of carbon, energy dispersive x‐ray spectroscopy (EDS) mapping was performed on CuO_250_, as shown in Figures S7 and S8 (Supporting Information). The results demonstrated the presence of carbon material in CuO_250_ with a high atomic percentage of 77.15%. This carbon structure altered the electronic structure of CuO_250_, resulting in a red shift of the XRD peaks. Second, the chemical composition and valence state of the material were further confirmed by XPS. The high‐resolution XPS spectrum of Cu 2p of CuO_500_ (Figure 2b) exhibited two peaks at 933.57 and 953.48 eV, which were attributed to Cu 2p_3/2_ and Cu 2p_1/2_ of Cu (+2). The peaks at binding energies of 941.35 and 943.78 eV can be recognized as the characteristic satellite peaks corresponding to Cu (+2). When comparing the binding energies at Cu 2p_3/2_ of CuO at different temperatures, it was observed that the binding energy shift of C‐containing CuO250 was the largest, followed by CuO_500_ (CuO_250_>CuO_500_>CuO_700_>CuO_400_>CuO_600_). This phenomenon was mainly attributed to the varying contents of oxygen vacancies formed on the material's surface on the surface at different calcination temperatures. The high‐resolution O 1s spectra (Figure S2, Supporting Information) showed peaks at 529.78, 531.66, 532.05, and 533.40 eV, corresponding to the Cu─O bond (OL), oxygen vacancy (OV), carbon‐oxygen bond (C═O), and adsorbed water molecules on the CuO surface (OW), respectively. The integration of the OV peak areas of CuO at different calcination temperatures resulted in an OV content order of CuO_500_>CuO_700_>CuO_400_>CuO_600_>CuO_250_, which was consistent with the Cu 2p_3/2_ binding energy shift results. Finally, we have characterized the morphology and structure of CuO_500_ using SEM and TEM. **Figure** **3**a–c illustrates CuO_500_, which is a spherical shape composed of small particles with an average size of 50 nm. High‐resolution transmission electron microscopy (HR‐TEM) (Figure 3d) confirmed the material's good crystallinity. Fourier transform analysis of the crystal surface revealed a lattice spacing of 0.25 nm, corresponding to the CuO (111) crystal surface, which was consistent with the XRD results. In conclusion, CuO was successfully synthesized at various calcination temperatures.

### 2.2 NO_3_RR Performance

**Figure 4:**
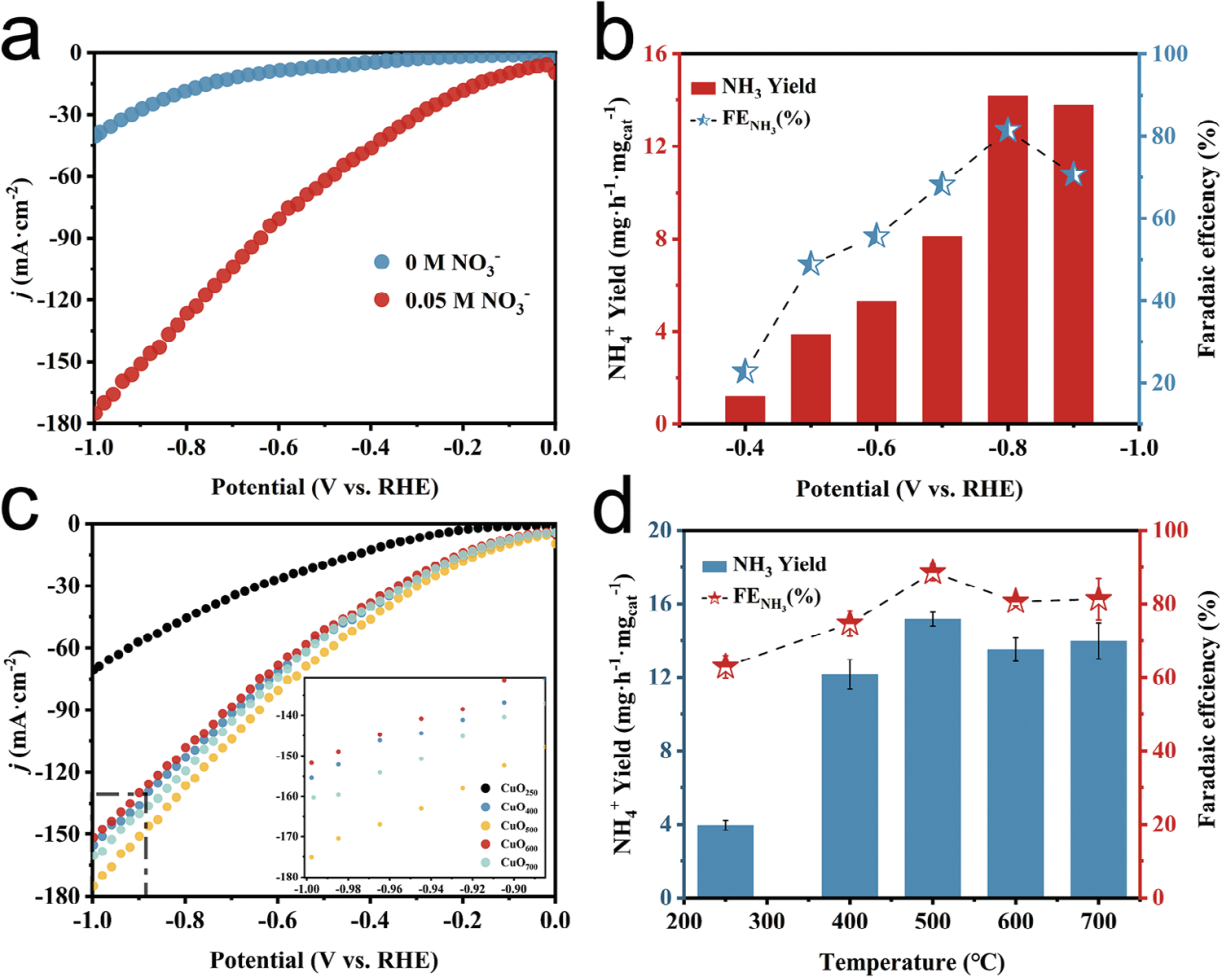
a) LSV curves with and without NO_3_^−^. b) Ammonia yield and FE after 2 h *i‐t* electrolysis of CuO_500_. c) LSV curves of CuO_250_ (black), CuO_400_ (blue), CuO_500_ (yellow), CuO_600_ (red), and CuO_700_ (blue–green). d) Ammonia yield and FE at −0.80 V after 1 h *i‐t* electrolysis of the material with different calcination temperatures.

The electrochemical performance of CuO/CC electrodes was evaluated in neutral electrolytes using a three‐electrode system in a Nafion 117 membrane‐separated H‐type electrolytic cell. Linear sweep voltammetry (LSV) scans were conducted in 0.5 M Na_2_SO_4_ electrolyte with and without NO_3_^−^ (0.05 M) at a scan rate of 10 mV/s (**Figure** **4**a). The addition of NO_3_^−^ significantly increased the current density at the same potential, indicating the occurrence of NO_3_RR on CuO/CC. The performance of CuO/CC was measured by continuous electrolysis for 2 h at a range of voltages from −0.40 to −0.90 V (vs RHE) to determine its NO_3_RR activity. NH_4_^+^ concentration in the electrolyte was determined by UV‐visible spectrophotometry and calibration curve. Figure 4b shows that the NH_3_ production rate increased gradually with the potential and peaked at −0.80 V (vs RHE), resulting in an ammonia yield of 14.2 mg h^−1^ mg_cat_^−1^ (or 835.34 umol h^−1^ mg_cat_^−1^). The optimum FE potential of the catalyst was also found at −0.80 V (vs RHE), with a FE of 81.42%. Therefore, we selected −0.80 V (vs RHE) to evaluate the continuous electrolysis measurements. The ammonia yield and FE of CuO/CC were higher than those of most NO_3_RR electrocatalytic catalysts reported in the literature (Table S1, Supporting Information).

To further understand the effect of calcination temperature on the catalytic properties, we synthesized catalysts with different calcination temperatures, labeled as CuO_250_, CuO_400_, CuO_500_, CuO_600_, and CuO_700_, by calcining at 250, 400, 500, 600, and 700 °C, respectively. Characterization results, including XPS and XRD, indicated successful synthesis of CuO at various calcination temperatures. SEM results showed that CuO at other calcining temperatures, except CuO_250_, formed a spherical structure with small particles stacked up, as shown in Figure S11 (Supporting Information). The integral analysis of the OV peak areas in Figure S2 (Supporting Information)’s high‐resolution O 1s spectra revealed that the peak areas followed the order of CuO_500_ > CuO_700_ > CuO_400_ > CuO_600_ > CuO_250_. The literature suggested that the presence of oxygen vacancies can alter the electronic structure of the electrode surface, leading to an increase in the adsorption of NO_3_^−^ and facilitating the decisive step of the reaction.^[^ ^28^ ^]^ This was well confirmed by the results of LSV scan (Figure 4c), where CuO_500_ with the highest content of oxygen vacancies exhibited the highest current density at the same potential, and the higher current density produced indicated the higher NO_3_RR performance. Then, materials with different calcination temperatures were subjected to 1 h *i‐t* tests. The results were presented in Figure 4d, which had the highest oxygen vacancy content, showing the best ammonia yield and FE of 15.53 mg h^−1^ mg_cat_^−1^ and 90.69%, respectively. Furthermore, due to the near absence of oxygen vacancies on the surface of CuO_250_ and the insufficient exposure of the copper oxide reaction sites surrounded by the carbon material, CuO_250_ displays markedly inferior performance compared to CuO_400_, CuO_500_, CuO_600_, and CuO_700_.These findings were consistent with the LSV scan. The increase in ammonia yield and FE at 1 h i‐t compared to 2 h *i‐t* was attributed to the decrease in the reaction rate caused by the reduction in NO_3_^−^ concentration in the system with increased electrolysis time.^[^ ^37^ ^]^

**Figure 5:**
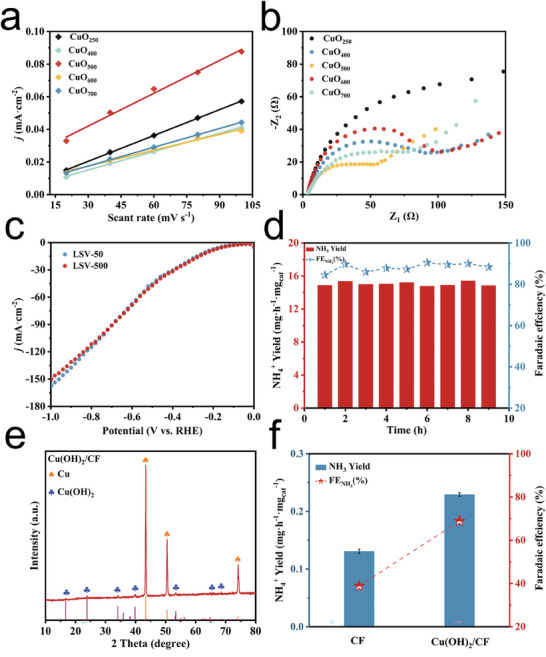
a) ECSA and b) EIS of CuO at different calcination temperatures. c) CV stabilization cycle test. d) Stability test of CuO/CC electrode at −0.80 V (vs RHE). e) XRD of Cu(OH)_2_/CF. f) NH_3_ yield and FE after 1 h electrolysis of CF and Cu(OH)_2_/CF.

The catalysts' electrochemically active surface area (ECSA) was determined by measuring the double‐layer capacitance (Cdl). According to **Figure** **5**a, the Cdl value of CuO_500_ was higher than that of the other CuO_x_, indicating that CuO_500_ has the largest ECSA. Furthermore, EIS measurements were further performed to evaluate the charge transfer resistance of the catalytic electrodes during the electrocatalytic NO_3_RR process. The Nyquist plot in Figure 5b showed that CuO_500_ exhibits a smaller resistance value, which was in line with its smaller semicircular diameter.

To assess the electrocatalytic stability of CuO/CC, continuous cyclic i‐t measurements were performed at −0.80 V (vs RHE) (Figure 5d). The ammonia yield and FE did not change significantly after each cycle, indicating good electrocatalytic stability. During the cyclic voltammetry (CV) cycling stability test, 500‐turn CV scans were conducted within the range of 0–1.0 V (vs RHE). The LSV curves obtained after the scans were found to be in agreement with the initial LSV curves (Figure 5c), indicating the excellent stability of CuO/CC. The XRD analyses of the electrodes after 1 and 5 h of electrolysis yielded identical results (Figure 7b), further confirming the stability of the electrode material.

### 2.3 Influence on NO_3_RR Performance of Electrode Phase and Structure Changes

**Figure 6:**
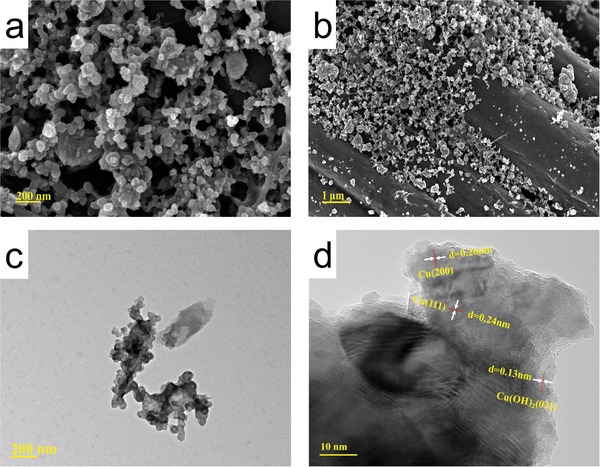
a,b) SEM, c) TEM, and d) HR‐TEM images of CuO_500_/CC after electrolysis.

**Figure 7:**
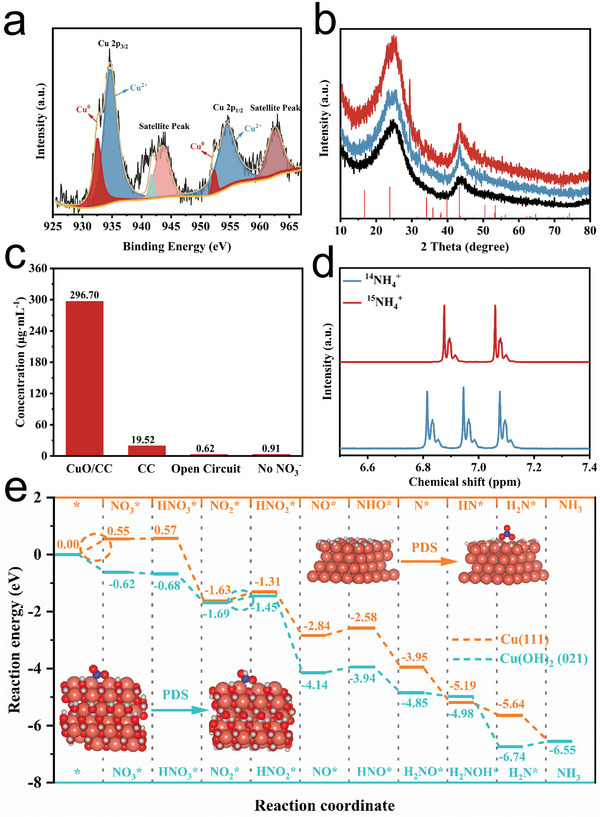
a,b) Characterizations of CuO_500_/CC after electrolysis: a) Cu 2p XPS and b) XRD after electrolysis for 1 h (red), 5 h (blue), and CC (black). c) NH_3_ concentration of CuO/CC in the presence or absence of NO_3_^−^, CC, and open potential conditions at −0.80 V (vs RHE). d) ^1^H‐NMR of ^14^NH_4_^+^ and ^15^NH_4_^+^, e) Calculated free energy diagram of NO_3_RR on Cu (111) and Cu(OH)_2_ (021).

During the i‐t test, we observed that a trace amount of blue material was generated on the electrode surface. Combined with the 1 and 5 h XRD data after electrolysis in the cyclic stability test, we speculated that the catalyst CuO may have undergone surface structure reconstruction during the NO_3_RR process. To investigate the catalyst's activity source, we conducted structural characterization of the CuO_500_/CC electrode after NO_3_RR treatment. The SEM and TEM images (**Figure** **6**a–c) showed a change in the surface morphology from the original stacked spherical morphology to a lamellar network structure of small particles stacked. The XRD spectra of the electrolyzed CuO electrode showed characteristic diffraction peaks at 43.3° and 50.4°, corresponding to the Cu (111) and (200) crystal planes, respectively, in addition to characteristic diffraction peaks at 16.7° and 23.8°, corresponding to Cu(OH)_2_ (PDF#80‐0656) (Figure 7b). This was in good agreement with the HR‐TEM crystal spacing measurement (Figure 6d). The XPS spectra consistently revealed the valence state of Cu on the surface of the reacted electrode, and in the high‐resolution XPS spectrum of Cu 2p (**Figure** **7**a), obvious satellite peaks at 941.71 and 943.55 eV can be clearly observed, suggesting that there was the presence of Cu (+2), and in combination with the peaks at the binding energy of Cu 2p_3/2_ at 934.68 eV (the binding energy of copper hydroxide was larger than that of copper oxide here). It can be determined that there was the presence of copper hydroxide. The peaks of Cu 2p_3/2_ and Cu 2p_1/2_ at 932.56 and 952.30 eV binding energies, respectively, corresponded to Cu (0). In summary, the real reactive site in the NO_3_RR process was Cu/Cu(OH)_2_.

The electrode's true active site was formed by the reconstruction of the CuO/CC electrode surface during the NO_3_RR process, with Cu/Cu(OH)_2_ being the active site. The monomeric copper was a product of Cu^2+^ reduction at the cathode; Cu(OH)_2_ was induced by the depletion of H^+^/H* during the NO_3_RR process resulting in a local pH increase and the production of strongly oxidizing *NO_2_ intermediates.^[^ ^38^, ^39^ ^]^ Therefore, the presence of oxygen vacancies on the initial electrode CuO/CC accelerated the initial NO_3_RR rate and the generation of *NO_2_ intermediates, and facilitated the conversion of CuO to the true active site of Cu/Cu(OH)_2_, which in turn promoted the overall NO_3_RR rate. Electrolytic measurements were conducted on CuO samples calcinated at various temperatures. The results indicated that CuO_500_ with the highest oxygen vacancies exhibited the highest ammonia yield and FE, confirming the previous observation. Meanwhile, CuO_500_, which had the highest oxygen vacancy content, demonstrated the highest ECSA and the lowest resistivity, indicating that it had higher reactivity and consumed fewer extra electrons. This was consistent with CuO_500_ showing the highest FE in the reaction, and likewise demonstrated that a higher content of oxygen vacancies at the initial electrode favored its conversion to the active site.

Additionally, we used CF and Cu(OH)_2_/CF as control electrodes to investigate the impact of component and structural modifications on the catalysts' performance during in situ reconstruction. Figure 5e shows the characteristic diffraction peaks of Cu (PDF#70‐3039) and Cu(OH)_2_ (PDF#80‐0656) detected on the Cu(OH)_2_/CF electrode, confirming successful synthesis of the Cu(OH)_2_/CF electrode. Electrolysis measurements were conducted for 1 h on CF and Cu(OH)_2_/CF, and the results were presented in Figure 5f. The ammonia yield of Cu(OH)_2_/CF (0.23 mg h^−1^ mg_cat_^−1^) was almost double that of CF (0.13 mg h^−1^ mg_cat_^−1^). The experimental results indicated that the introduction of Cu(OH)_2_ improved the performance of NO_3_RR. During the experiment, more bubbles were observed on the electrode surface of CF at the same voltage, while almost no bubbles were generated on the surface of the Cu(OH)_2_/CF electrode. This suggested that the introduction of copper hydroxide can effectively inhibit the HER side reaction, which was also well verified by the higher FE of Cu(OH)_2_/CF. Comparing the performance of Cu(OH)_2_/CF with that of CuO_500_/CC, the difference in performances between the two was large, indicating that the micro‐size structure of the electrode also played an important role in the catalytic process.

### 2.4 Ammonia Sources

To verify that the obtained ammonia was obtained by electrocatalytic nitrate reduction reaction, a series of comparative experiments were performed, which were electrochemically tested at 0.5 m Na_2_SO_4_ + 0.05 m NO_3_^−^, 0.5 m Na_2_SO_4_, blank CC, and open circuit potential conditions for 1 h. The results, as shown in Figure 7c, showed almost no NH_3_ production in the absence of nitrate and at open circuit voltage. The yield of blank CC was also only one‐fifteenth of that of CuO/CC. It was shown that NH_3_ was obtained by electrolysis of CuO/CC electrode in the presence of 0.5 m Na_2_SO_4_ + 0.05 m NO_3_^−^.

In addition, to further verify that the product ammonia in the NO_3_RR process was all derived from the reactant nitrate, we carried out isotopic labeling analysis using ^15^NO_3_^−^ and ^14^NO_3_^−^, and conducted a 2 h *i‐t* electrolysis using ^15^NO_3_^−^ and ^14^NO_3_^−^ as reactants, respectively. Following electrolysis, the electrolyte from the cathode was collected and 2 M H_2_SO_4_ was added until the pH reached 3. The solution was then concentrated to 1–2 mL in an oven. Then ^1^H‐NMR was taken and the results are shown in Figure 7d. The electrolyte with electrocatalytic reduction of ^15^NO_3_^−^ showed typical doublet peaks of ^15^NH_4_^+^ at 6.88 and 7.06 ppm, and the electrolyte with ^14^NO_3_^−^ showed typical triplet peaks of ^14^NH_4_^+^ at 6.81, 6.95, and 7.08 ppm in the ^1^H‐NMR spectra. This experiment confirmed that the formation of the product ammonia originated from the electroreduction of nitrate.

### 2.5 DFT Theoretical Analysis

Here, DFT calculations were also employed to elucidate the enhanced catalytic performance originating from the reconstructed Cu/Cu(OH)_2_. Prior to exploring the mechanism of NO_3_RR, the surface Pourbaix diagram of the Cu (111) catalyst was investigated under the experimental conditions. As the result shown in Figure S12 (Supporting Information), the most stable surface state of Cu (111) under experimental potential was fully covered by the hydrogen generated from the solution. Therefore, the following analysis of reaction mechanism of NO_3_RR on Cu (111) focused on a H‐covered surface. Several possible reaction pathways for NO_3_RR were explored on Cu (111) and Cu(OH)_2_ (021). The most energetically favorable pathway on Cu (111) was identified to be NO_3_*→HNO_3_*→NO_2_*→HNO_2_*→NO*→NHO*→N*→HN*→H_2_N*→NH_3_. On Cu(OH)_2_ (021), the preferred pathway was found to involve the following intermediates: NO_3_*→HNO_3_*→NO_2_*→HNO_2_*→NO*→HNO*→H_2_NO*→H_2_NOH*→H_2_N*→NH_3_.

Nitrate adsorption on the catalysts plays a crucial role in triggering the NO_3_RR. As depicted in Figure 7e, the free energy diagrams reveal a clear difference in the thermodynamic favorability of the NO_3_RR initial step, that is, nitrate adsorption. On the Cu(OH)_2_ (021) surface, this step exhibited an exothermic reaction energy of −0.62 eV, indicating a spontaneous and energetically favorable process. In contrast, on the Cu (111) surface, nitrate adsorption requires an endothermic energy ≈ 0.55 eV, which was regarded as the potential determining step (PDS) for the entire NO_3_RR process on Cu (111). However, the potential determining step (PDS) of NO_3_RR on Cu(OH)_2_ (021) was identified as the fourth step, that is, the protonation of NO_2_*, with a corresponding reaction energy of 0.24 eV. The calculated lower reaction energy of the PDS strengthened the experimental observation that the improved catalytic performance of NO_3_RR was attributed to the reconstruction leading to the formation of Cu(OH)_2_.

## 3 Conclusion

In summary, copper oxide spheres with an average particle size of 50 nm stacked with small particles were synthesized by a simple sol‐gel method as an efficient electrocatalyst for the synthesis of ammonia by electrochemical NO_3_RR. The electrochemical measurements showed that the CuO/CC cathode exhibited excellent NO_3_RR performance, achieving an NH_3_ yield of 15.53 mg h^−1^ mg_cat_^−1^ (or 913.53 umol h^−1^ mg_cat_^−1^) and a FE of 90.69%. Meanwhile, our experimental data and DFT calculations suggested that the high activity of the CuO/CC electrode was due to the reforming of the surface phase and structure during electrolysis. In the electrocatalytic process, the structural change increased the number of reactive sites and facilitated the mass/charge transfer process. The conversion of CuO to Cu/Cu(OH)_2_ in the phase and the reconstructed multivalent (0, +2) composition enhanced the electron transfer for the reaction at the electrode surface. The Cu crystal surface generated by the reduction facilitates the hydrogenation reaction, and the presence of Cu(OH)_2_ enhanced the adsorption of nitrate and the inhibition of the HER. The synergistic effect of both showed excellent catalytic performance in NO_3_RR. In addition, this study provided new insights and ideas for using copper‐based oxide catalysts in NO_3_RR.

## 4 Experimental Section

### Chemicals and Reagents

Triblock poly(ethylene oxide)‐b‐poly(propylene oxide)‐b‐poly(ethylene oxide) Pluronic F127 (EO_106_PO_70_EO_106_, Mav = 12600, Pluronic F127) and citric acid (C_6_H_8_O_7_ 99.5%) were purchased from McLean. Nitric acid (HNO_3_ 98%), n‐butanol (CH_3_(CH_2_)_3_OH 99.5%), copper nitrate trihydrate (Cu(NO_3_)_2_·3H_2_O 99.2%), ethanol (C_2_H_5_OH 99.7%), sodium persulfate ((NH_4_)_2_S_2_O_8_ 99.9%), and sodium hydroxide (NaOH 95%) were purchased from the National Pharmaceutical Chemical Reagent Co. Ltd. Sodium hypochlorite solution (NaClO ≥ 5%), sodium nitroprusside dihydrate, and Nafion solution (5 wt%) were purchased from Aladdin. Copper foam (CF) was purchased from Suzhou Kesheng and Metal Materials. All the chemical reagents were used as is without further purification.

### Preparation of CuO Catalyst

Pluronic F127 (0.79 g) and C_6_H_8_O_7_ (0.96 g) were dissolved in CH_3_(CH_2_)_3_OH (6.41 mL) by sonication to form a homogeneous solution. HNO_3_ (0.72 mL) was then slowly added to form a transparent solution, labelled as solution A. Cu(NO_3_)_2_·3H_2_O (0.60 g) was added to solution A and dissolved with stirring to form an azure blue solution, labelled as solution B. Solution B was then spread flatly in a petri dish and evaporated at 120 °C for 6 h in a drying oven. The gel labelled as gel A was transferred to a vacuum drying oven and dried at 80 °C for 12 h. Subsequently, the gel labelled as A underwent calcination in a muffle furnace at 150 °C (heating rate: 2 °C min^−1^) for 6 h, followed by 6 h at 250, 400, 500, 600, and 700 °C (heating rate: 2 °C min^−1^) each. The result was five material sets labeled CuO_250_, CuO_400_, CuO_500_, CuO_600_, and CuO_700_.

### Preparation of CF and Cu(OH)_2_/CF Catalyst

The CF electrode was prepared by immersing a 1 × 1 cm^2^ sheet of CF into 3 m HCl and cleaning it ultrasonically for 20 min. It was then washed alternately with ethanol and water three times before being dried in a vacuum oven at 60 °C for 12 h.

The cleaned CF was immersed in a 100 ml aqueous solution containing (NH_4_)_2_S_2_O_8_ (0.125 m) and NaOH (2.5 m) and left to stand under ambient conditions for 30 min. After the reaction, the samples were removed from the solution, washed with deionized water, and dried in a vacuum drying oven at 60 °C for 12 h to obtain the Cu(OH)_2_/CF electrode.

### Characterizations

The morphology of the prepared catalyst samples was characterized using field emission scanning electron microscopy (FE‐SEM, GeminiSEM 300, Zeiss) and transmission electron microscopy (TEM, JEM‐2100, Nippon Electron) at 200 KV; the surface of the materials was irradiated using X‐rays in an X‐ray diffractometer (XRD, D8 ADVANCE, Bruker), the information about the crystal structure of the material, Cu Kα radiation (λ1/4 = 1.5418 Å), was calculated by collecting the diffraction signals. The composition and elemental valence information of the samples were obtained by X‐ray photoelectron spectroscopy (XPS, ESCALAB XI+, Thermo Fisher). The absorbance of the solution after electrolysis was measured in a UV‐1800 UV spectrophotometer to estimate the NH_3_ yield. The isotopes ^14^NH_4_^+^, ^15^NH_4_^+^ were measured using nuclear magnetic resonance hydrogen spectroscopy (^1^H‐NMR, 400MR, Agilent).

### Electrochemical Measurements

A Koster electrochemical workstation (CS310M) was used for the electrochemical tests. The NO_3_RR electrochemical measurements were evaluated with a standard three‐electrode system, where the counter electrode was Pt foil, the reference electrode was Ag/AgCl, and the working electrode was self‐prepared as follows.

Prior to preparing the CuO/CC working electrode, the carbon cloth (CC) underwent ultrasonic cleaning three times with deionized water and ethanol for 5 min each time to eliminate surface impurities. Subsequently, it was dried under vacuum at 50 °C for 12 h. To prepare the CuO/CC working electrode, 5 mg of CuO was dissolved in 0.99 mL of a mixed solution of ethanol and water (50/49) using ultrasound. 0.01 mL of Nafion (5 wt%) solution was added to the above solution to form a uniform ink. 0.1 mL of the prepared ink was applied uniformly to the pretreated CC (1 × 1 cm^2^) and dried at 50 °C to form a working electrode.

Electrochemical studies of NO_3_RR were carried out in a two‐compartment H‐type electrolytic cell separated by a Nafion 117 membrane, with 25 mL of 0.5 m Na_2_SO_4_ (containing 0.05 m NO_3_^−^) solution in the cathode compartment and 25 mL of 0.5 m Na_2_SO_4_ solution in the anode compartment. The electrolyte underwent purification using argon (99.99%) for 30 min before undergoing electrochemical tests. Chronoamperometry(*i‐t*) tests were conducted at varying applied potentials with a constant stirring rate. Electrochemical impedance spectroscopy (EIS) measurements were performed in a 0.5 M aqueous Na_2_SO_4_ solution with 0.05 m NO_3_^−^ at a voltage of −0.2 V (vs RHE), a frequency of 100 kHz to 0.1 Hz, and an amplitude of 5 mV. All potentials measured in this study were converted to the RHE reference potentials for comparison. The RHE reference potentials were calculated using the following formula: *E_RHE_* (*V*) = *E_REF_* (*V*) + *E*_*Vs*.*Ag*/*AgCl*_ + 0.059*pH.

## Conflict of Interest

The authors declare no conflict interest.

## Supporting information

Supporting Information

## Data Availability

The data that support the findings of this study are available from the corresponding author upon reasonable request.
