# Supplementary material for: Surface Structure Reformulation from CuO to Cu/Cu(OH)2 for Highly Efficient Nitrate Reduction to Ammonia
Source: Adv Sci (Weinh). 2024 Aug 9;11(38):2404194. doi: 10.1002/advs.202404194 (PMC11481209; doi:10.1002/advs.202404194)
Supplement: Supplementary file 1 — Supporting Information [file ADVS-11-2404194-s001.docx]

**Supporting Information**

**Surface Structure Reformulation From CuO to Cu/Cu(OH)_2_ for Highly Efficient Nitrate Reduction to Ammonia**

Jin Li^a^, Qiuling Jiang^b,d,e^, Xiujing Xing^c^, Cuilian Sun^a^, Ying Wang^e^, Zhijian Wu^d,e^, Wei Xiong^a,^*^*^*, Hao Li^b,*^

*^a^ Key Laboratory of Novel Biomass-Based Environmental and Energy Materials in Petroleum and Chemical Industry, Key Laboratory of Green Chemical Engineering Process of Ministry of Education, Hubei Key Laboratory of Novel Reactor &Green Chemical Technology, School of Chemistry and Environmental Engineering, Wuhan Institute of Technology, Wuhan 430205, China*

*^b^ Advanced Institute for Materials Research (WPI-AIMR), Tohoku University, Sendai 980-8577, Japan*

*^c^ Chemistry Department, University of California, Davis, California 95616, United States*

*^d^ School of Applied Chemistry and Engineering, University of Science and Technology of China, Hefei 230026, China*

*^e^ Changchun Institute of Applied Chemistry, Chinese Academy of Sciences, Changchun 130022, China*

Corresponding author

E-mail address: xiongwei@wit.edu.cn (W. X.); li.hao.b8@tohoku.ac.jp (H. L.)

Tel/Fax: +86-27-87195001; +81-080-9363-8256​

Detection of ammonia

The indophenol blue method was used to measure ammonia from cathodic reduction. The electrolyte was first diluted to the detection range after electrolysis. Then, a mixture of 1 M NaOH, salicylic acid (5 wt%), and sodium citrate trihydrate (5 wt%) was added to 2 mL of the diluted sample. Sequentially, 1 mL of 0.05 M sodium hypochlorite solution and 0.2 mL of aqueous sodium nitroprusside (1 wt%) were added. The mixture was allowed to stand for 1 hour at room temperature, protected from light. The absorbance at 653 nm was then recorded using a UV spectrometer. The standard curve for determining ammonia by the indophenol blue method was obtained using a series of (NH_4_)_2_SO_4_ standards dissolved in a 0.50 M Na_2_SO_4_ solution. The equation y = 0.3386x + 0.0429 (R^2^ = 0.999) was used to calculate the concentration of NH_3_ in the unknown samples (Fig. S1a, b). The NH_3_ yield and FE were then calculated using the following equations:

NH_3_ yield rate (μg h^−1^ mg_cat_^−1^) = $\frac{c_{NH_{3}}\times V}{m\times t}$ (1)

FE (%) = $\frac{8\times F\times c_{NH_{3}}\times v}{17\times Q}\times100\%$ (2)

where c_NH3_ (μg mL^-1^) is the measured concentration of NH_3_ in the electrolyte, V (mL) is the volume of the electrolyte, t (h) is the time of the electroreduction reaction, m (mg) is the effective weight of the catalyst at the working electrode, F is the Faraday's constant (96485 C mol^-1^), and Q is the applied electricity (C) in the reaction.


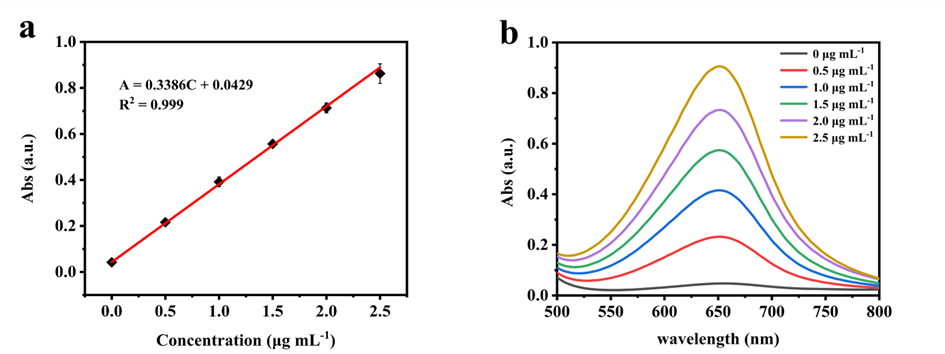


**Figure S1.** (a) UV-vis absorption spectra obtained using the indophenol blue method at different NH_3_ concentrations and (b) it’s fitting curve.


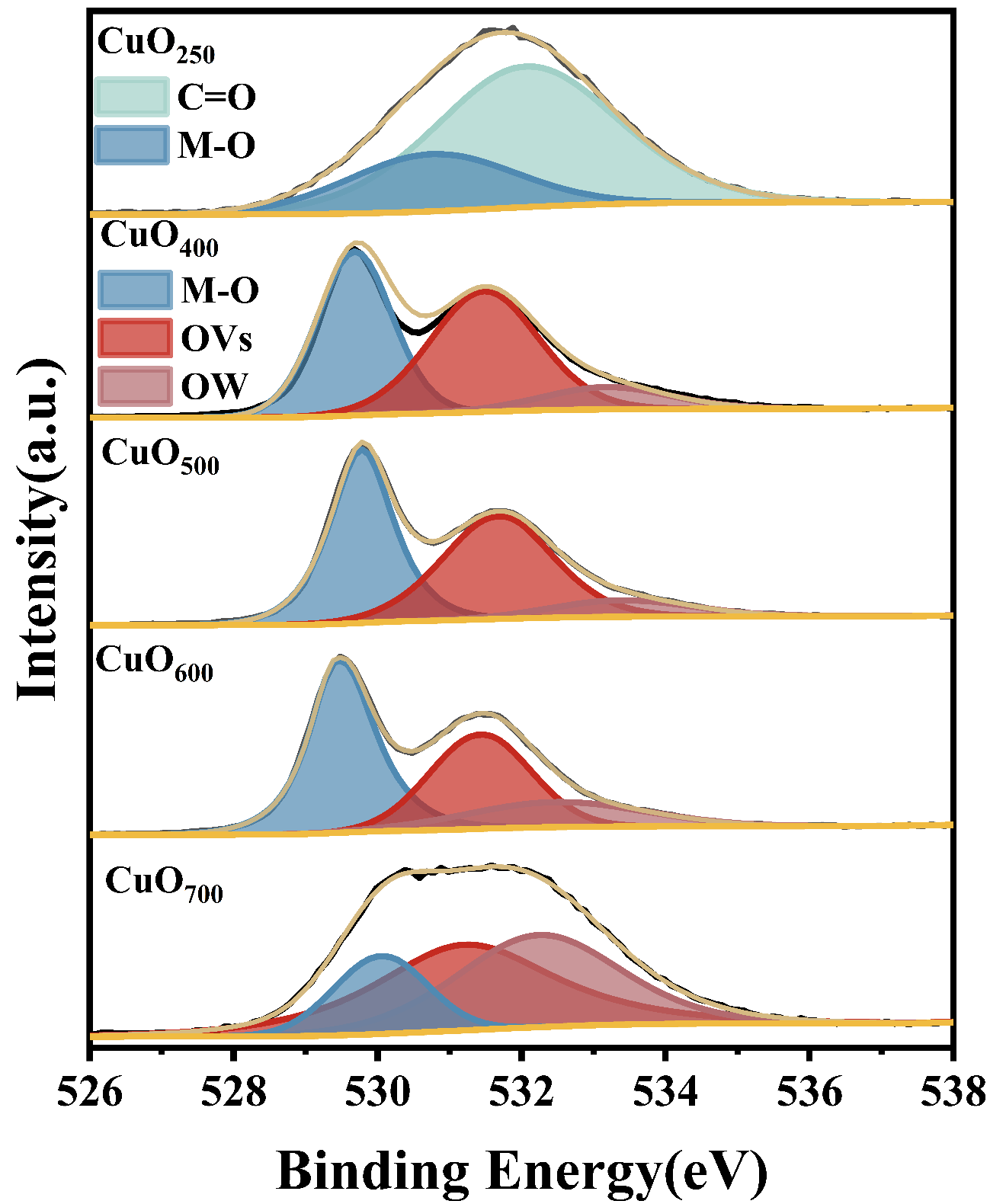


**Figure S2.** High-resolution XPS spectra of O 1s.


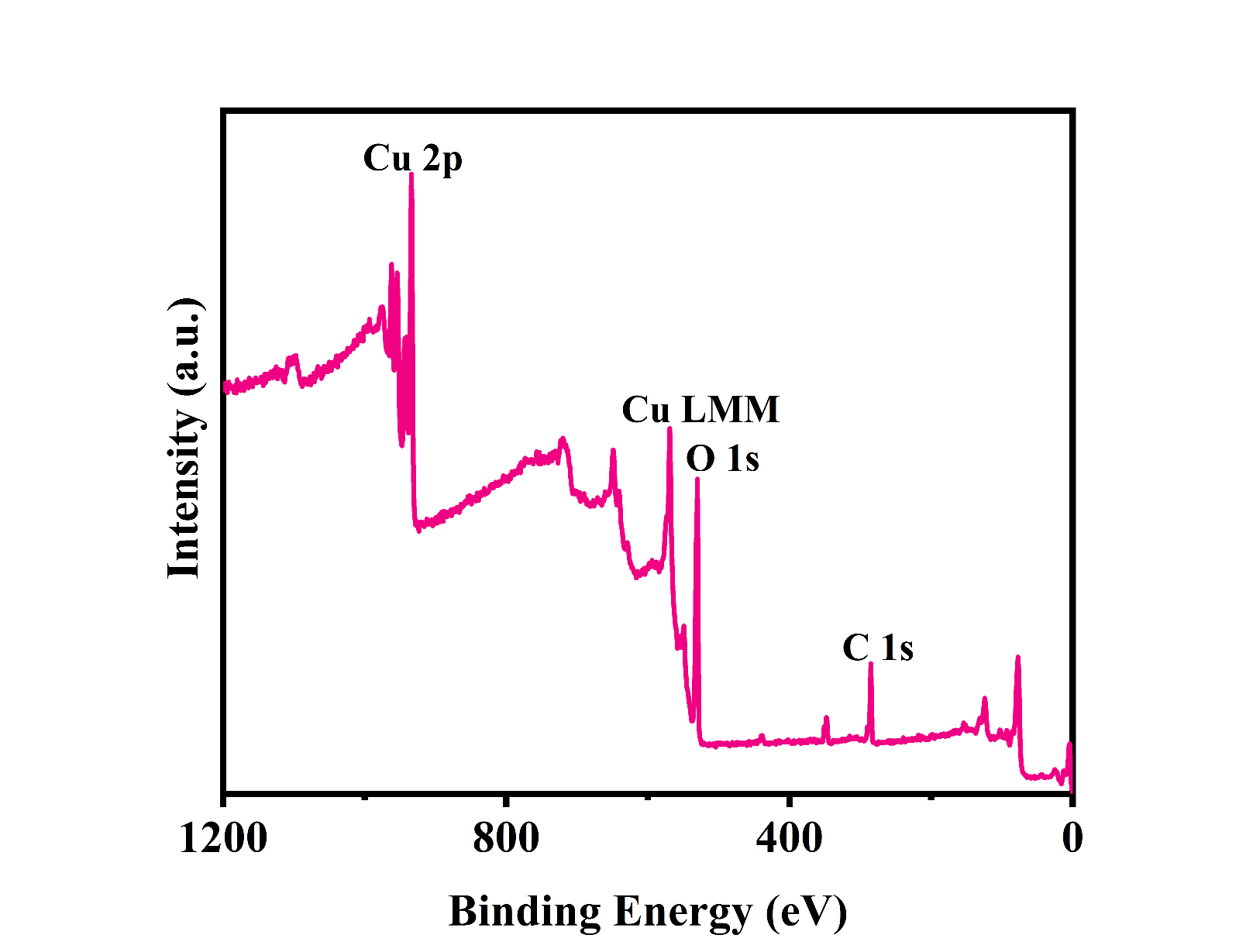


**Figure S3.** XPS full spectrum of CuO_500_.

**
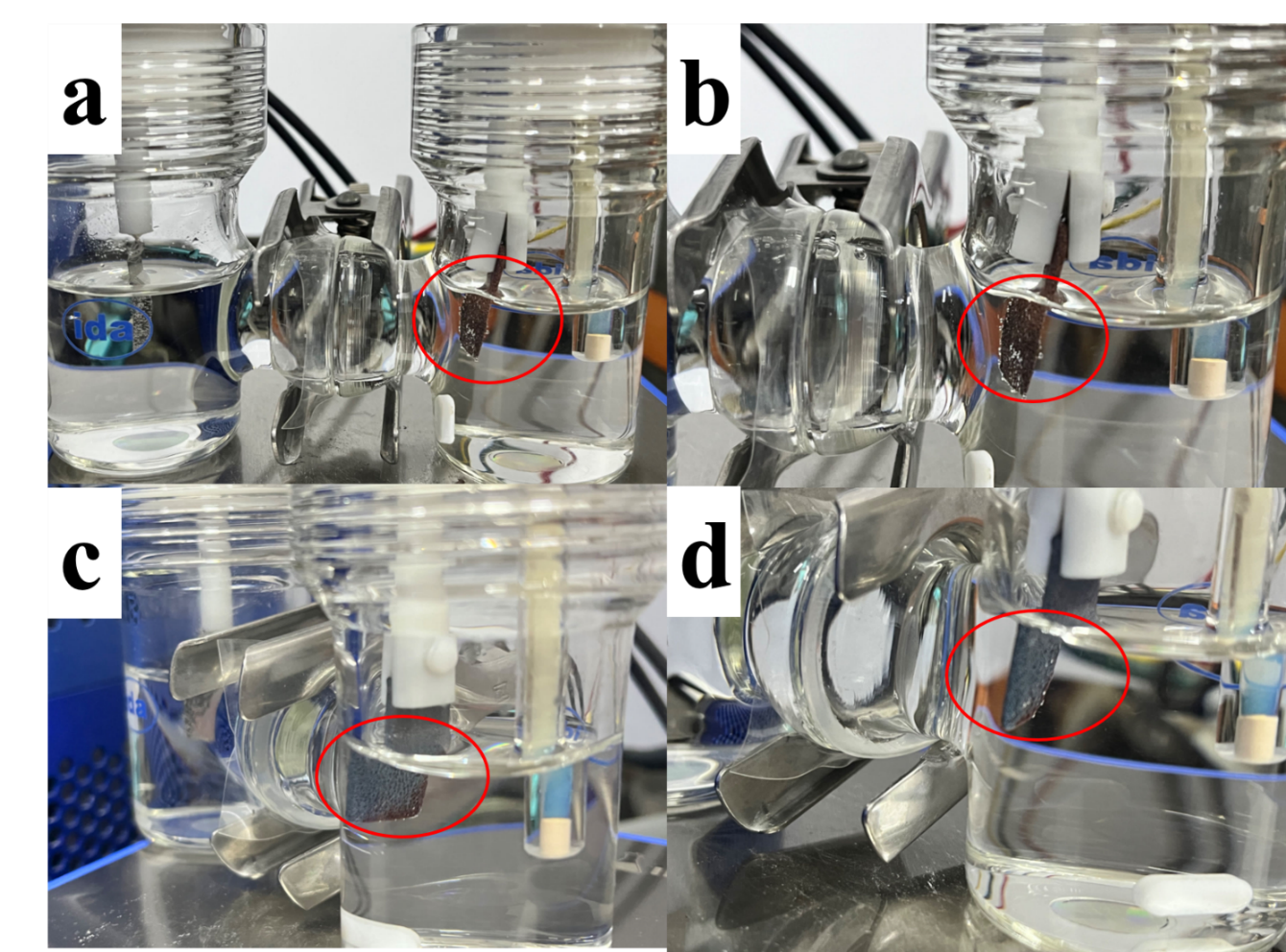
**

**Figure S4.** (a-b) Electrode surface condition during electrolysis of CF, (c-d) Electrode surface condition during electrolysis of Cu(OH)_2_/CF.


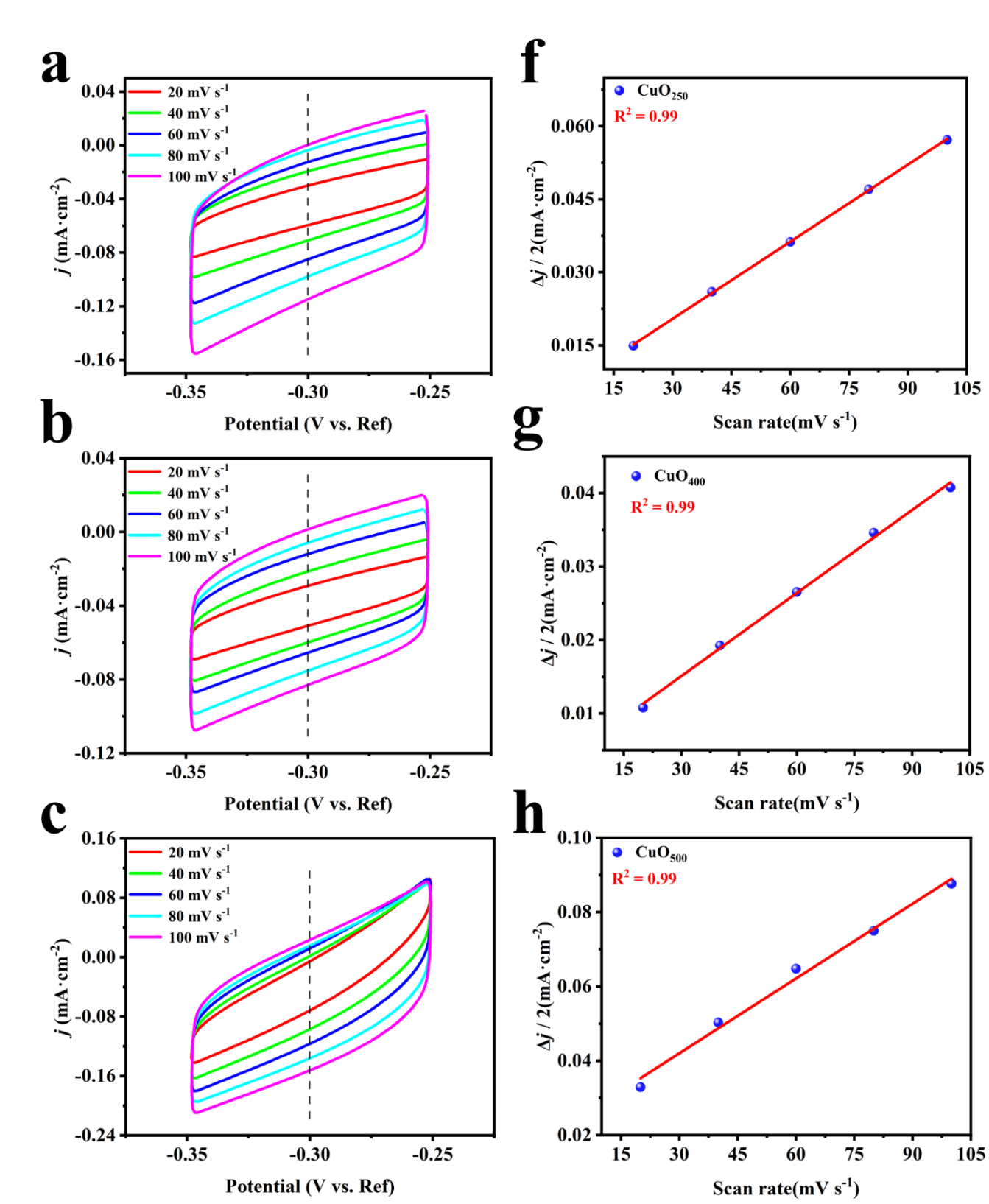


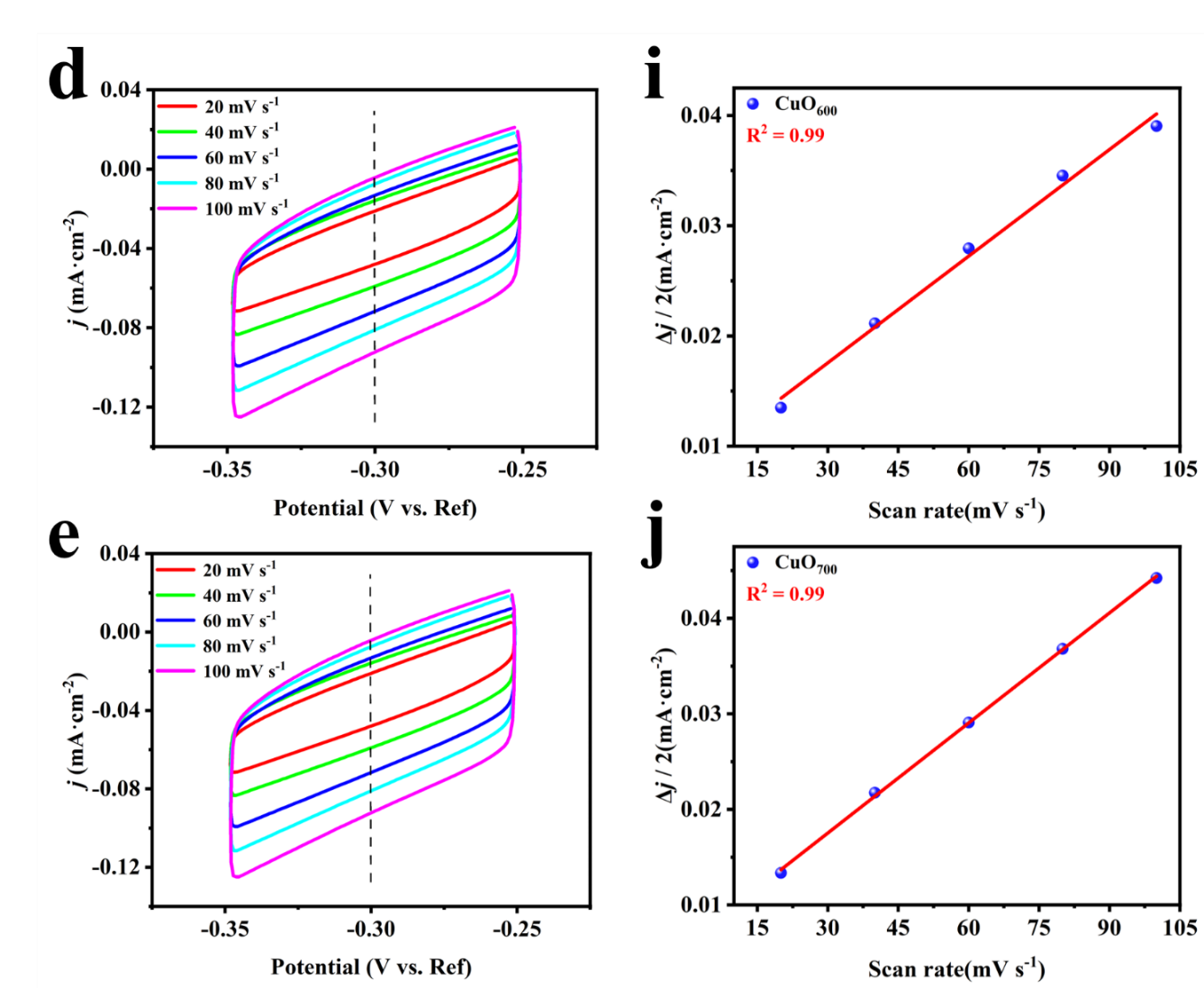


**Figure S5.** The CV data used to determine the ECSA. (a-e) CV in the non-faradaic region and (f-j) the capacitive current differences, Δ*j*/2, as a function of the CV scan rates of CuO_250_ (a, f), CuO_400_ (b, g), CuO_500_ (c, h), CuO_600_ (d, i) and CuO_700_(e, j).

**
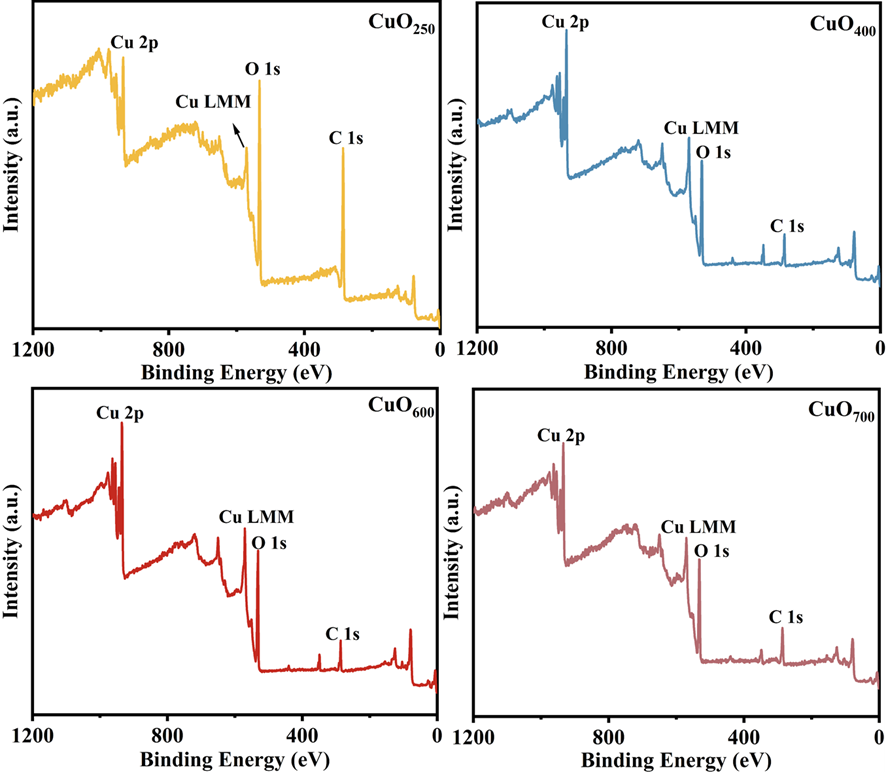
**

**Figure S6.** XPS full spectrum of CuO_250_, CuO_400_, CuO_600_, CuO_700_.

**
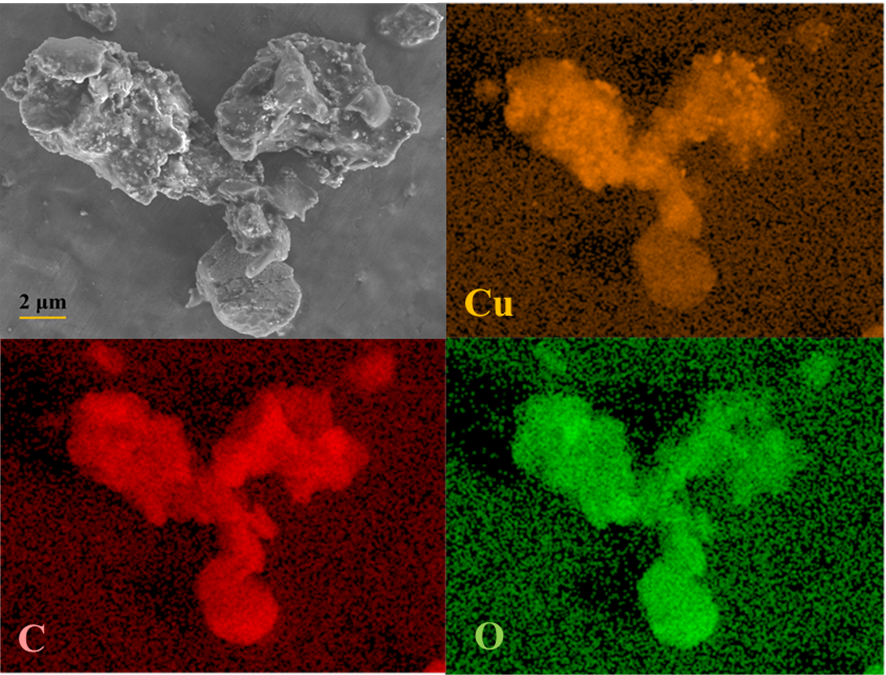
**

**Figure S7.** HAADF-STEM image and elemental mapping images of CuO_250._

**
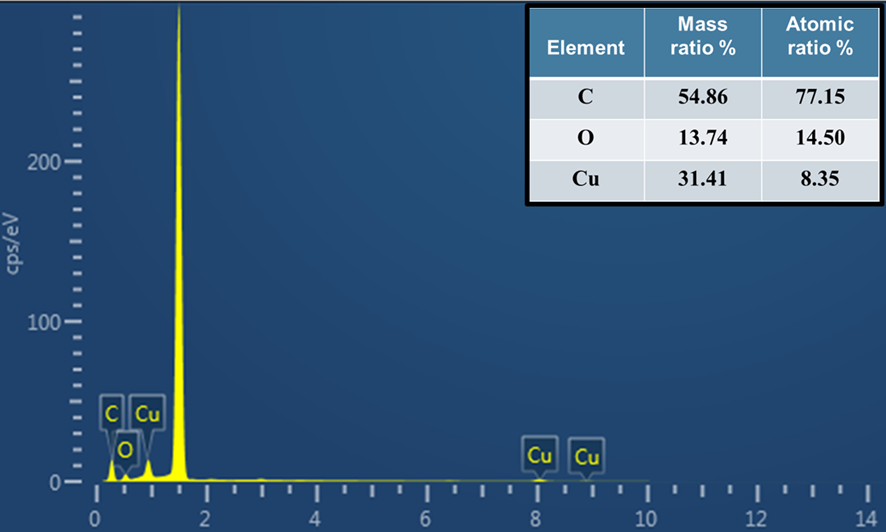
**

**Figure S8.** EDS spectrum of the CuO_250._

**
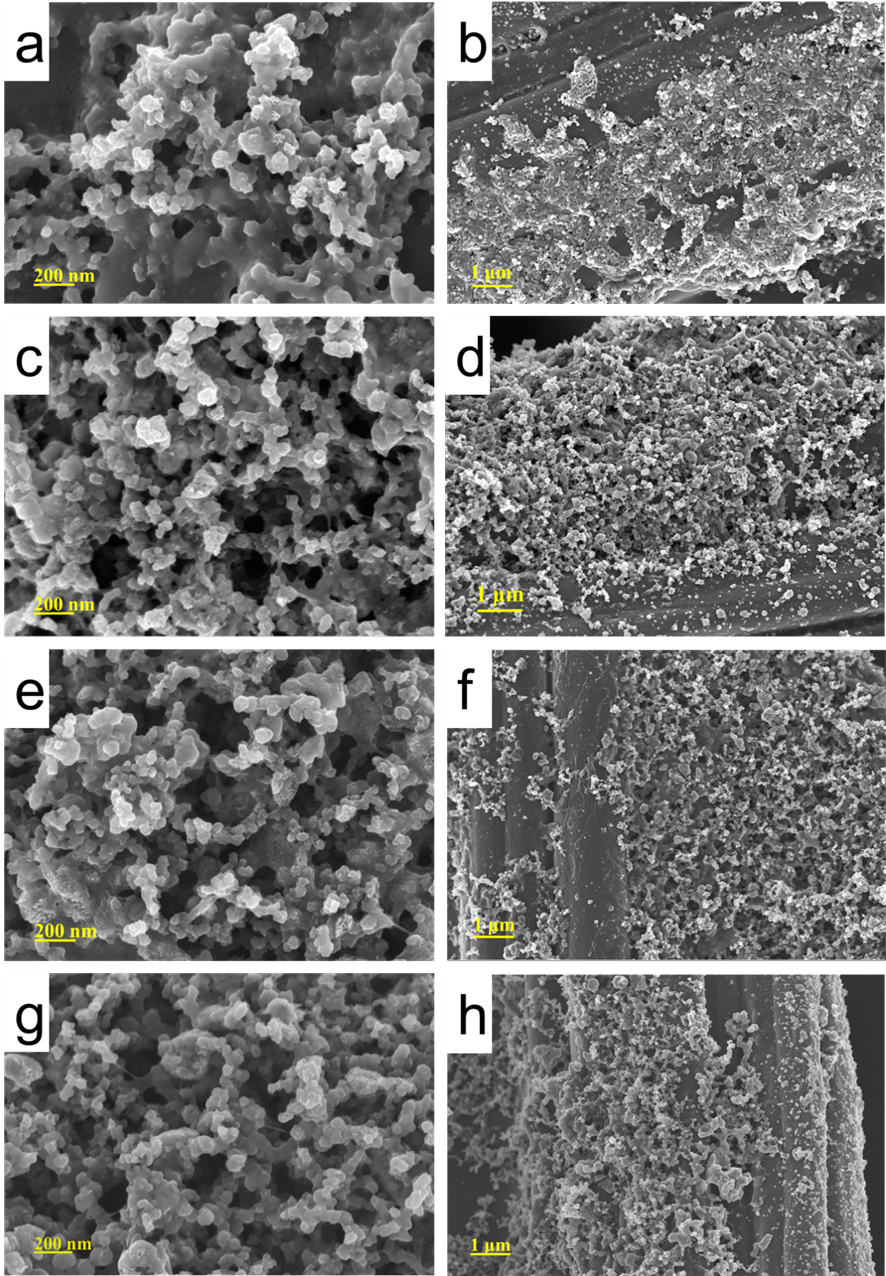
**

**Figure S9.** (a-b), (c-d), (e-f), (g-h) are SEM images of CuO_250_, CuO_400_, CuO_600_, CuO_700_ after electrolysis.

**
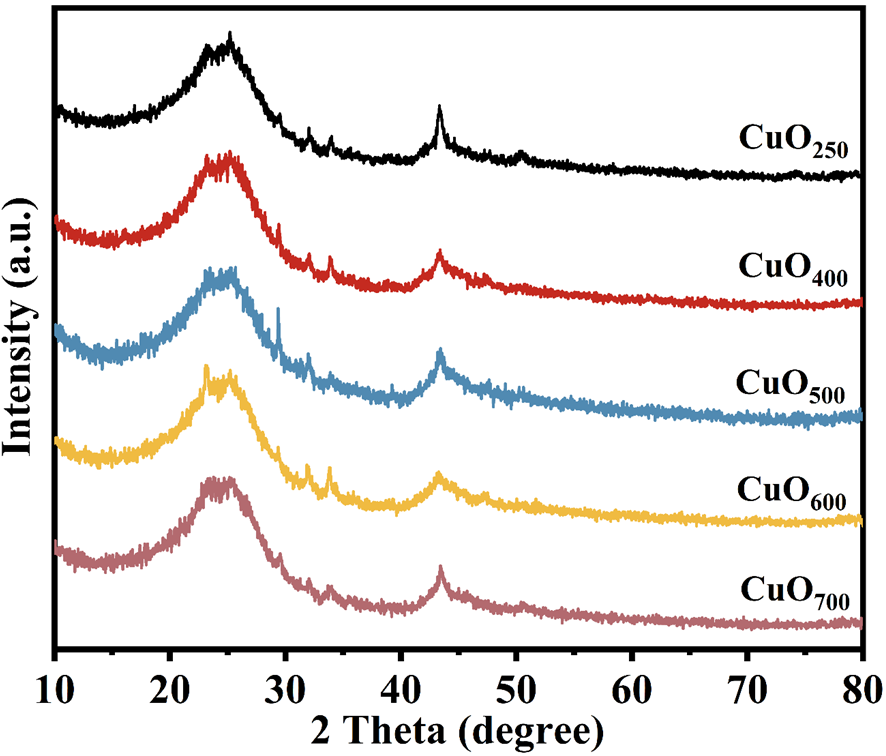
**

**Figure S10.** XRD of CuO_250_, CuO_400_, CuO_500_, CuO_600_, and CuO_700_ after one hour of electrolysis.

**
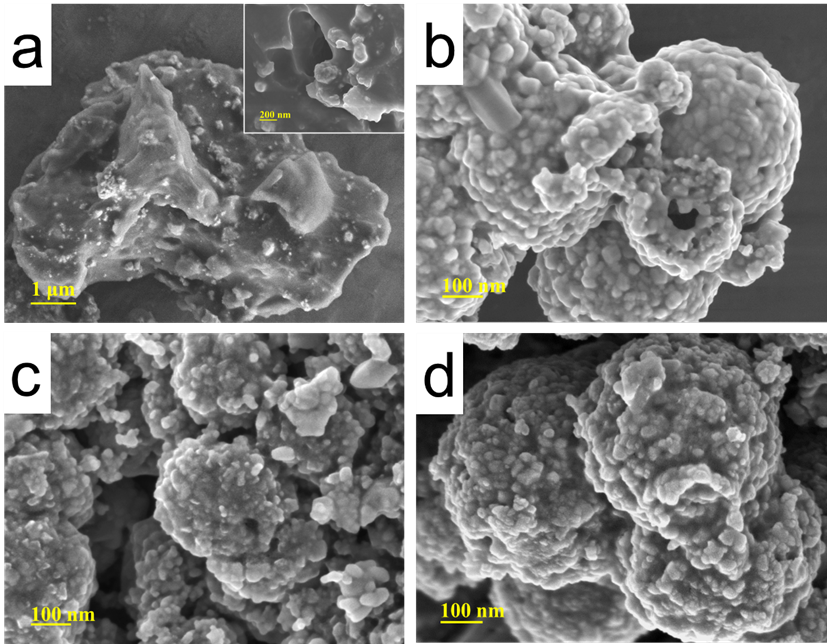
**

**Figure S11.** a, b, c, d are SEM images of CuO_250_, CuO_400_, CuO_600_, CuO_700._

**Computational details**

The Vienna ab initio simulation package (VASP) was used to perform the spin-polarized density functional theory (DFT) calculations ^[1]^. The revised Perdew-Burke-Ernzerhof (RPBE) functional with the generalized gradient approximation method was chosen to characterize the exchange and correlation interactions within the electronic system ^[2,3]^. A plane-wave basis set was specified with a cutoff of 400 eV. The projector-augmented wave (PAW) potentials were employed to describe the interaction between ions and electrons ^[4]^. The Brillouin zone was sampled with a Monkhorst-Pack 3 × 3 × 1 k-point mesh grid. The thresholds for force and energy convergence were 0.05 eV/Å and 10^–5^ eV, respectively. For the geometric model, the four-layer Cu (111) and Cu(OH)_2_ (021) supercells with a sufficient vacuum gap of 15 Å were built to simulate the exposed surfaces in this study. The bottom two layers of the Cu (111) slab were fixed during the structural relaxation.

The surface Pourbaix diagrams in this work were developed based on the computational hydrogen electrode (CHE) model as a function of pH and potential ^[5,6]^ . According to a generalized representation of the adsorption of oxygenated intermediates:

$H_{n}O_{m}^{*}+\left( 2m-n \right)\left( H^{+}+e^{-} \right)\rightleftarrows*+{mH}_{2}O$ (1)

where m and n are respectively the number of oxygen and hydrogen atoms adsorbed on the catalyst surface, the free energies of each surface state in the surface Pourbaix diagrams ($\Delta G(U,pH)$) can be calculated as follows:

$\Delta G(U,pH)=G_{bare}+mG_{H_{2}O}-G_{tot}-(2m-n)(\frac{1}{2}G_{H_{2}}-U_{SHE}-2.303k_{B}T*pH)$ (2)

where $G_{bare}$ and $G_{tot}$ are the total energies of the clean surface and surface with adsorbed oxygen and hydrogen atoms, respectively. $U_{SHE}$ is the potential referred to standard hydrogen electrode (SHE), $k_{B}$ is the Boltzmann constant, and $T$ sets to 298 K.

According to the calculation results, the favored reaction pathways for nitrate reduction to ammonia (NO_3_RR) in this study were as follows:

$NO_{3}^{-}+ H^{+}+ * \to{NO}_{3}^{*}+ H^{+}+ e^{-}$ (1)

$NO_{3}^{*}+H^{+}+ e^{-} \to{NO}_{3}H^{*}$ (2)

${NO}_{3}H^{*}+H^{+}+e^{-} \to NO_{2}^{*}+ H_{2}O$ (3)

$NO_{2}^{*}+H^{+}+ e^{-} \to{NO}_{2}H^{*}$ (4)

${NO}_{2}H^{*}+ H^{+}+ e^{-} \to{NO}^{*}+ H_{2}O$ (5)

${NO}^{*}+H^{+}+ e^{-} \to H{NO}^{*}$ (6.1)

${NO}^{*}+H^{+}+ e^{-} \to{NHO}^{*}$ (6.2)

$H{NO}^{*}+ H^{+}+ e^{-} \to{{NH}_{2}O}^{*}$ (7.1)

${NHO}^{*}+ H^{+}+ e^{-} \to N^{*}+H_{2}O$ (7.2)

${{NH}_{2}O}^{*}+H^{+}+ e^{-} \to{{NH}_{2}OH}^{*}$ (8.1)

$N^{*}+ H^{+}+ e^{-} \to{NH}^{*}$ (8.2)

${{NH}_{2}OH}^{*}+H^{+} + e^{-} \to{NH}_{2}^{*}+ H_{2}O$ (9.1)

${NH}^{*}+H^{+} + e^{-} \to{NH}_{2}^{*}$ (9.2)

${NH}_{2}^{*}+H^{+}+ e^{-} \to{NH}_{3}+ *$ (10)

Where the *** denotes the adsorbed site on the catalyst surface. The reaction energy of each step (*ΔG*) is calculated based on the computational hydrogen electrode CHE model ^[6]^, and defined by the following equation:

$$\Delta G=\Delta E+\Delta ZPE-T\Delta S$$

Where *ΔE* is the energy difference between the reactants and products. *∆ZPE* and *T∆S* correspond the zero-point energy correction and entropy change, respectively.

Surface Pourbaix diagram of Cu (111)
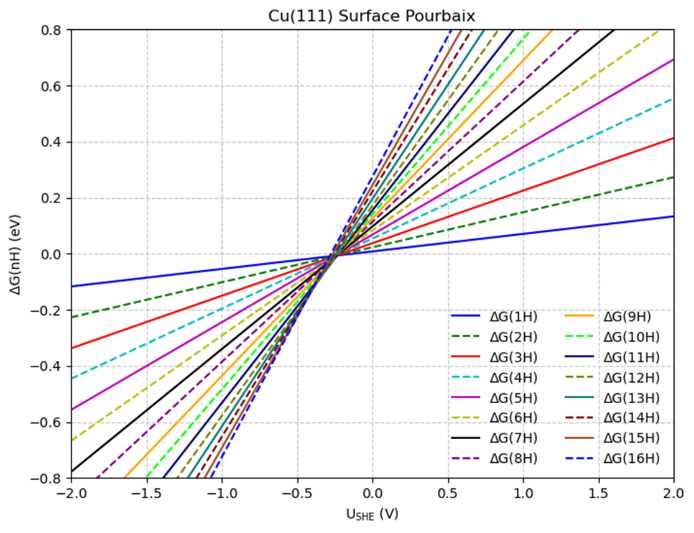


**Figure S12.** Calculated surface Pourbaix diagrams of Cu (111) as a function of pH and potential.

Geometric structures of intermediates in NO_3_RR on Cu (111) and Cu(OH)_2_ (021).


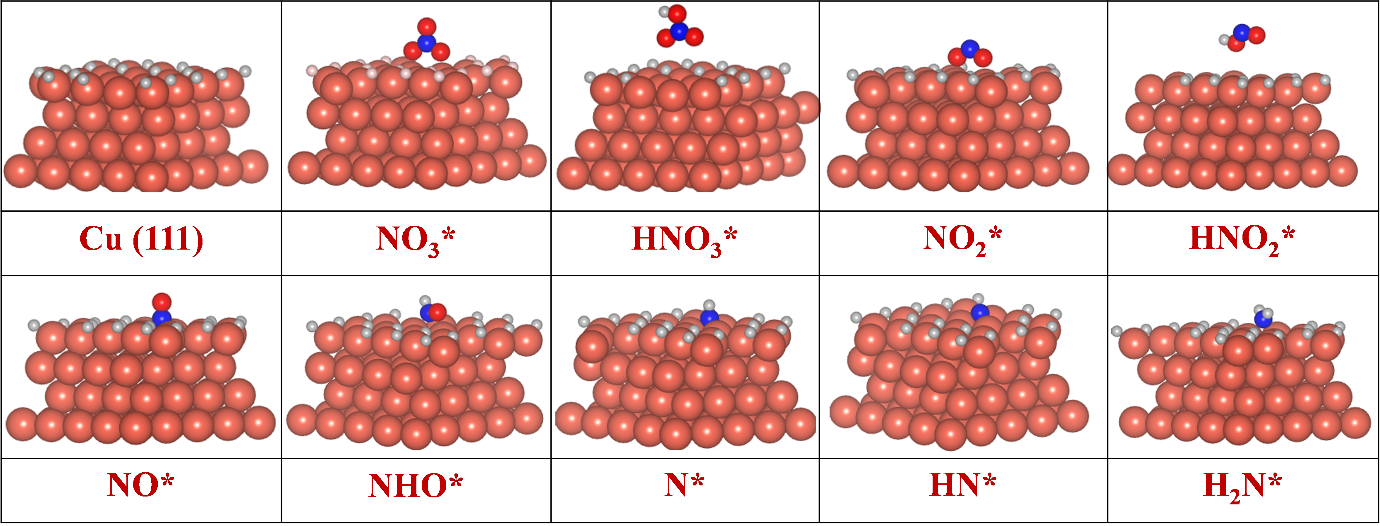


**Figure S13**. Optimized geometric structures of NO_3_RR intermediates on Cu (111).


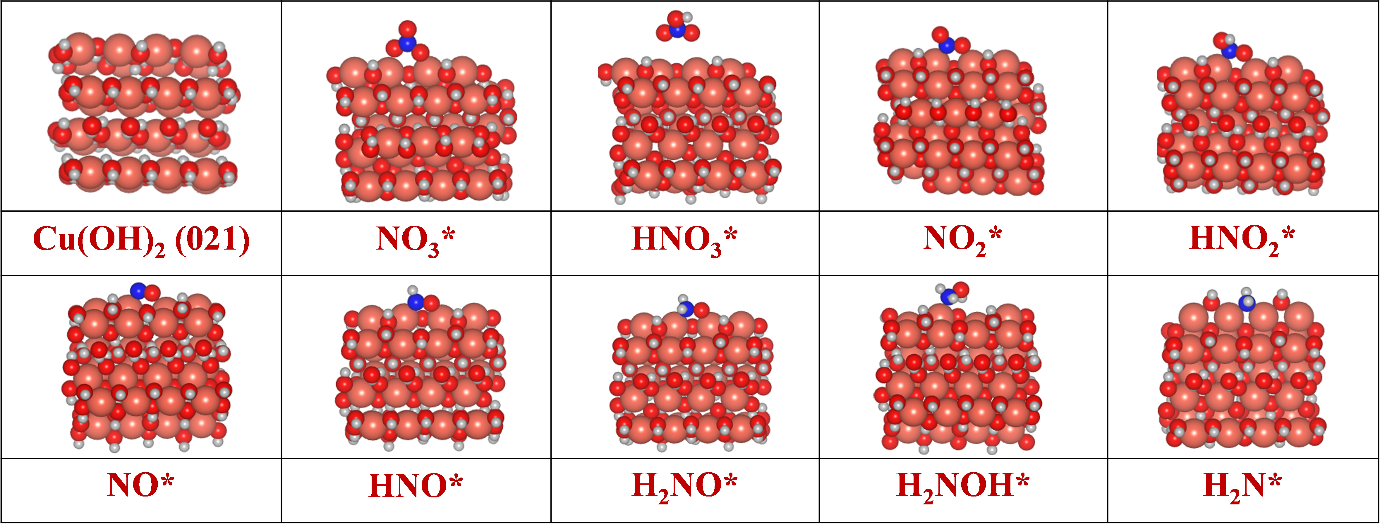


**Figure S14.** Optimized geometric structures of NO_3_RR intermediates on Cu(OH)_2_ (111).

**Table S1.** Comparison of catalyst performance**
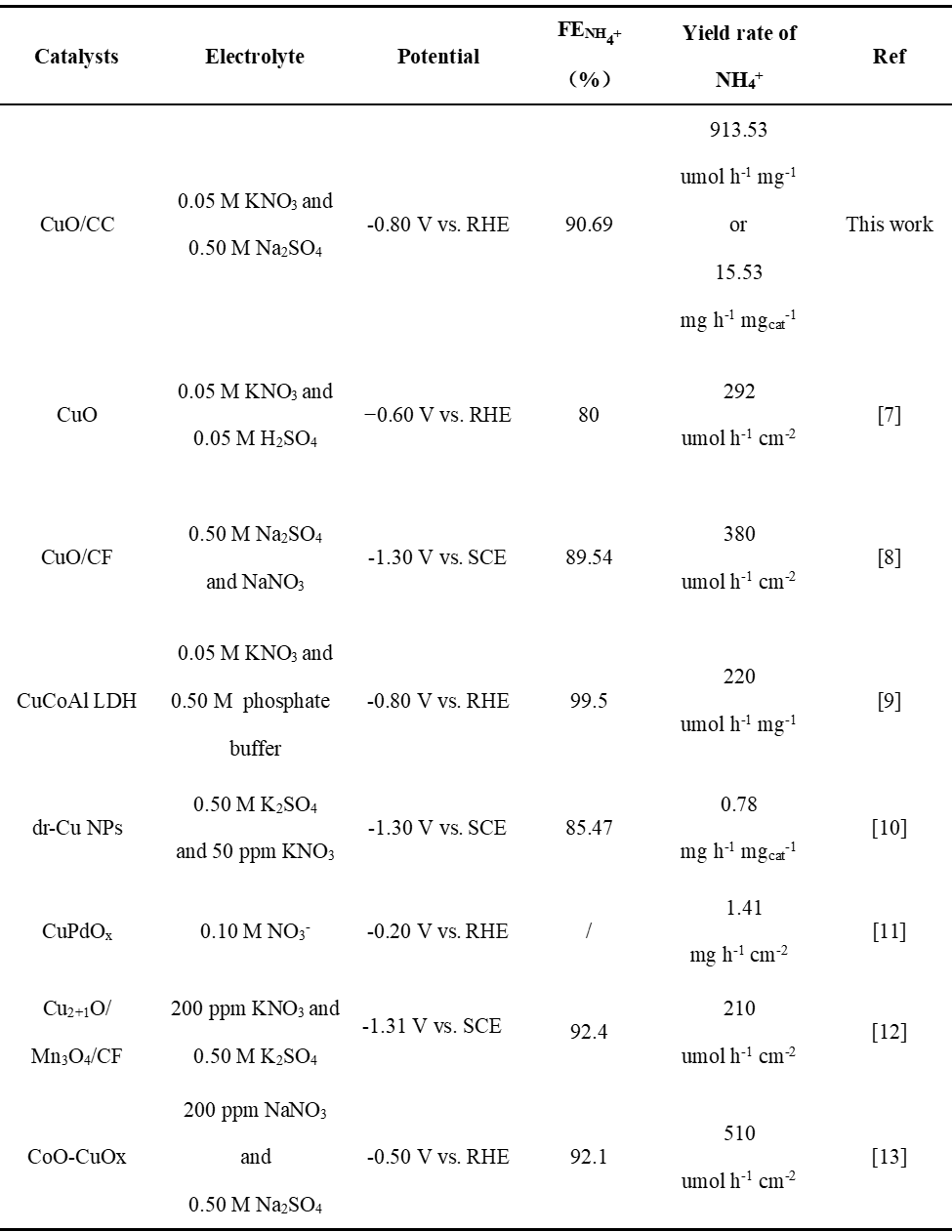
**

**Supplementary References**.

[1] Hafner J. Ab‐initio simulations of materials using VASP: Density‐functional theory and beyond. *Journal of computational chemistry*, **2008**, *29*(13): 2044-2078.

[2] Hammer B, Hansen L B, Nørskov J K. Improved adsorption energetics within density-functional theory using revised Perdew-Burke-Ernzerhof functionals. *Physical review B*, **1999**, *59*(11): 7413.

[3] Perdew J P, Burke K, Ernzerhof M. Generalized gradient approximation made simple. *Physical review letters*, **1996**, *77*(18): 3865.

[4] Blöchl P E. Projector augmented-wave method. *Physical review B*, **1994**, *50*(24): 17953.

[5] Hansen H A, Rossmeisl J, Nørskov J K. Surface Pourbaix diagrams and oxygen reduction activity of Pt, Ag and Ni (111) surfaces studied by DFT. *Physical Chemistry Chemical Physics*, **2008**, *10*(25): 3722-3730.

[6] Nørskov J K, Rossmeisl J, Logadottir A, et al. Origin of the overpotential for oxygen reduction at a fuel-cell cathode. *The Journal of Physical Chemistry B*, **2004**, *108*(46): 17886-17892.

[7] Daiyan R, Tran-Phu T, Kumar P, et al. Nitrate reduction to ammonium: from CuO defect engineering to waste NO_x_-to-NH_3_ economic feasibility. *Energy & Environmental Science*, **2021**, *14*(6): 3588-3598.

[8] Fang L, Wang S, Song C, et al. Boosting nitrate electroreduction to ammonia via in situ generated stacking faults in oxide-derived copper. *Chemical Engineering Journal*, **2022**, *446*: 137341.

[9] Wang W, Chen J, Tse E C M. Synergy between Cu and Co in a layered double hydroxide enables close to 100% nitrate-to-ammonia selectivity. *Journal of the American Chemical Society*, **2023**, *145*(49): 26678-26687.

[10] Xu Y, Wang M, Ren K, et al. Atomic defects in pothole-rich two-dimensional copper nanoplates triggering enhanced electrocatalytic selective nitrate-to-ammonia transformation. *Journal of Materials Chemistry A*, **2021**, *9*(30): 16411-16417.

[11] Jung W, Jeong J, Chae Y, et al. Synergistic bimetallic CuPd oxide alloy electrocatalyst for ammonia production from the electrochemical nitrate reaction. *Journal of Materials Chemistry A*, **2022**, *10*(44): 23760-23769.

[12] Jung W, Jeong J, Chae Y, et al. Synergistic bimetallic CuPd oxide alloy electrocatalyst for ammonia production from the electrochemical nitrate reaction. *Journal of Materials Chemistry A*, **2022**, *10*(44): 23760-23769.

[13] Tang Y, Liu S, Guo C, et al. Constructing a CoO–CuOx heterostructure for efficient electrochemical reduction of nitrate to ammonia. *Sustainable Energy & Fuels*, **2023**, *7*(20): 5039-5045.
